# Supplementary material for: Short-term safety and efficacy of aspirin in patients with COVID-19: a systematic review and meta-analysis of randomized controlled trials
Source: PeerJ. 2025 May 21;13:e19466. doi: 10.7717/peerj.19466 (PMC12103164; doi:10.7717/peerj.19466)
Supplement: Supplemental Information 3 [file peerj-13-19466-s003.doc]

| **sTable 2. Sensitivity analysis of main outcomes excluding one study at a time.** | | | | | | | | | | | | | | |
| --- | --- | --- | --- | --- | --- | --- | --- | --- | --- | --- | --- | --- | --- | --- |
|  | ACTIV-4B | | ACT-C | | ACT-H | | RECOVERY | | REMAP-CAP | | REMAP-CAP-n | | RESIST | |
|  | RR(CI) | P | RR(CI) | P | RR(CI) | P | RR(CI) | P | RR(CI) | P | RR(CI) | P | RR(CI) | P |
| **All-cause mortality** | 0.96 | 0.24 | 0.95 | 0.22 | 0.94 | 0.16 | 0.94 | 0.48 | 0.97 | 0.38 | NA | | 0.96 | 0.26 |
|  | (0.89, 1.03) |  | (0.88, 1.03) |  | (0.87, 1.02) |  | (0.80, 1.11) |  | (0.89, 1.04) |  |  |  | (0.89, 1.03) |  |
| **Bleeding** | 1.74 | <0.0001 | NA | | 1.66 | 0.0003 | 2.66 | 0 | 1.64 | 0.0003 | 1.71 | <0.0001 | 1.71 | <0.0001 |
|  | (1.33, 2.28) |  |  |  | (1.26, 2.19) |  | (1.40, 5.04) |  | (1.25, 2.15) |  | (1.32, 2.23) |  | (1.32, 2.23) |  |
| **Any thrombosis** | 0.89 | 0.08 | 0.88 | 0.07 | 0.88 | 0.07 | 0.92 | 0.57 | 0.87 | 0.05 | 0.88 | 0.06 | NA | |
|  | (0.77, 1.01) |  | (0.77, 1.01) |  | (0.77, 1.01) |  | (0.67, 1.24) |  | (0.75, 1.00) |  | (0.77, 1.01) |  |  |  |
| **High-flow oxygen or ventilation** | NA | | NA | | 0.95 | 0.29 | 0.87 | 0.87 | NA | | NA | | 0.93 | 0.15 |
|  |  |  |  |  | (0.85, 1.05) |  | (0.70, 1.08) |  |  |  |  |  | (0.85, 1.02) |  |
| **Discharged alive.** | NA | | NA | | NA | | 1.2 | 0.15 | 1.07 | 0.08 | 1.07 | 0.05 | NA | |
|  |  |  |  |  |  |  | (0.94, 1.54) |  | (0.99, 1.15) |  | (1.00, 1.15) |  |  |  |
| **AKI** | NA | | 1 | 0.96 | 1 | 0.97 | 1.53 | 0.37 | NA | | NA | | NA | |
|  |  |  | (0.85, 1.19) |  | (0.84, 1.18) |  | (0.61, 3.85) |  |  |  |  |  |  |  |
| AKI=acute kidney injury, NA=not applicable | | | | | | | | | | | | | | |
