# Supplementary material for: Short-term safety and efficacy of aspirin in patients with COVID-19: a systematic review and meta-analysis of randomized controlled trials
Source: PeerJ. 2025 May 21;13:e19466. doi: 10.7717/peerj.19466 (PMC12103164; doi:10.7717/peerj.19466)
Supplement: Supplemental Information 4 [file peerj-13-19466-s004.doc]

**Appendix Table S2. Full electronic search strategy through June 30, 2024.**

Ovid MEDLINE(R) and Epub Ahead of Print, In-Process, In-Data-Review & Other Non-Indexed Citations, Daily and Versions <1946 to June 30, 2024>

1 exp aspirin / 48077

2 acetylsalicylic acid.mp. 10558

3 acetylsalicylate.mp. 630

4 Anti-platelet.mp. 5778

5 exp COVID-19/ 198258

6 exp SARS-CoV-2/ 152181

7 1 or 2 or 3 or 4 56981

8 5 or 6 200739

9 7 and 8 204

Embase <1974 to June 30, 2024>

1 Aspirin.mp. 126859

2 acetylsalicylate.mp. 2408

3 exp coronavirus disease 2019/ 288232

4 SARS-CoV-2.mp. 130340

5 1 or 2 128440

6 3 or 4 321714

7 5 and 6 643

Duplucated =182

Cochrane Library：

Search Name:

Date Run: 06/30/2024 22:40:15

#1 (Aspirin) OR (acetylsalicylic acid) OR (acetylsalicylate) OR ("anti-platelet") (Word variations have been searched) 18970

#2 (COVID-19) OR (SARS-CoV-2) 13839

#3 #1 AND #2 75
