# Supplementary figures and images for: Short-term safety and efficacy of aspirin in patients with COVID-19: a systematic review and meta-analysis of randomized controlled trials
Source: PeerJ. 2025 May 21;13:e19466. doi: 10.7717/peerj.19466 (PMC12103164; doi:10.7717/peerj.19466)

**sFigure 1. Risk of bias assessment of included trials.**


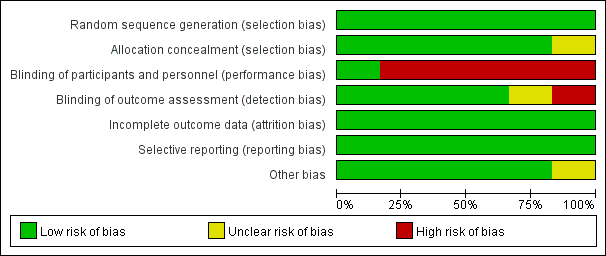

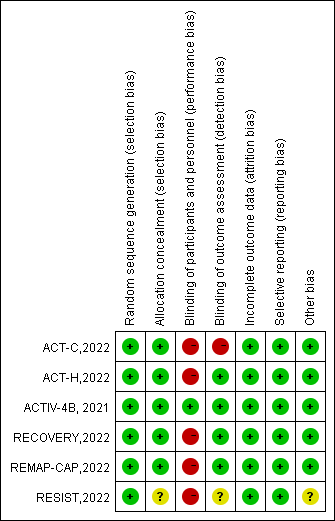

Supplement: Supplemental Information 5 [file peerj-13-19466-s005.doc]
