# Supplementary material for: Short-term safety and efficacy of aspirin in patients with COVID-19: a systematic review and meta-analysis of randomized controlled trials
Source: PeerJ. 2025 May 21;13:e19466. doi: 10.7717/peerj.19466 (PMC12103164; doi:10.7717/peerj.19466)

sFigure 2. Forest plots of asprin treatment vs. non-asprin treatment for the bleeding (A: major bleeding; B: minor bleeding). CI = confidence intervals; M-H = Mantel-Haenszel; IV= inverse variance


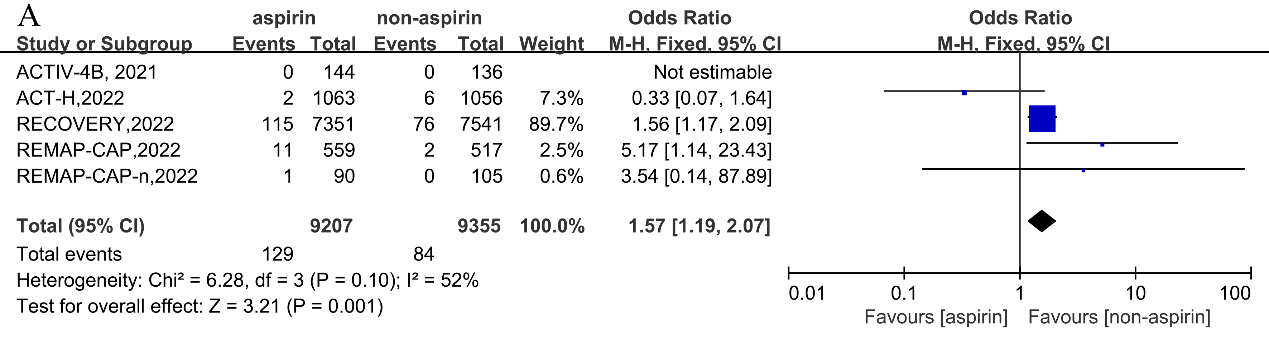


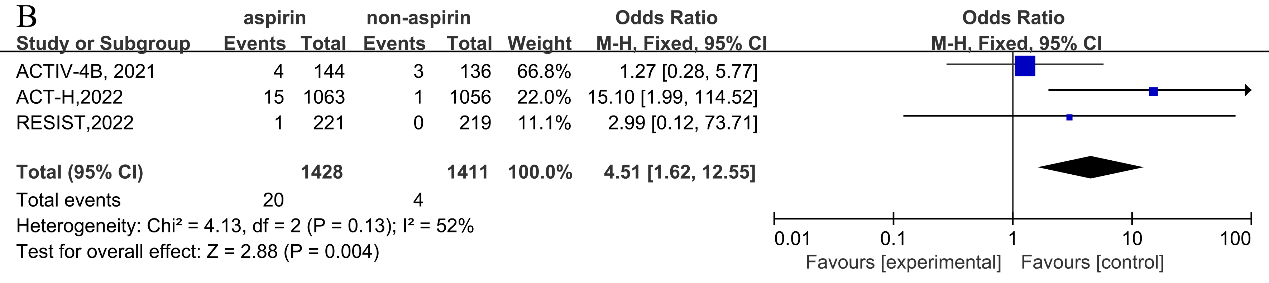


sFigure 3. Forest plots of asprin treatment vs. non-asprin treatment for outcomes (A: deep venous thrombosis; B: pulmonary embolism; C: stroke; D: myocardial infarction). CI= confidence intervals; M-H= Mantel-Haenszel; IV= inverse variance


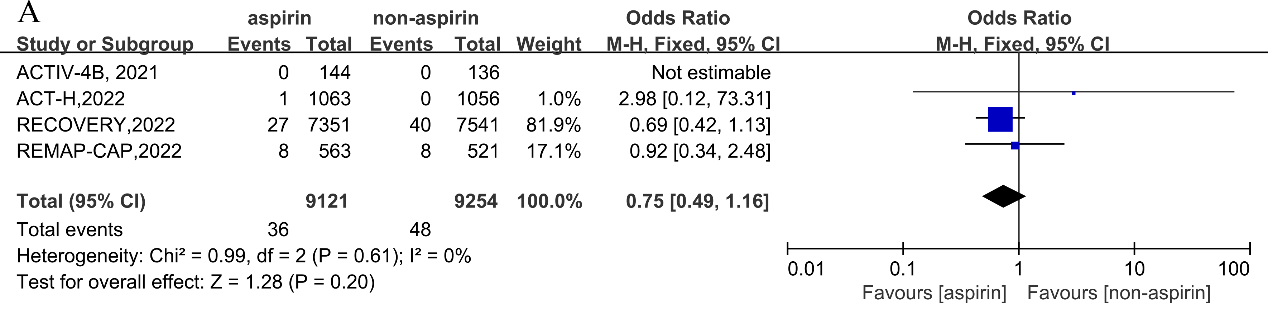


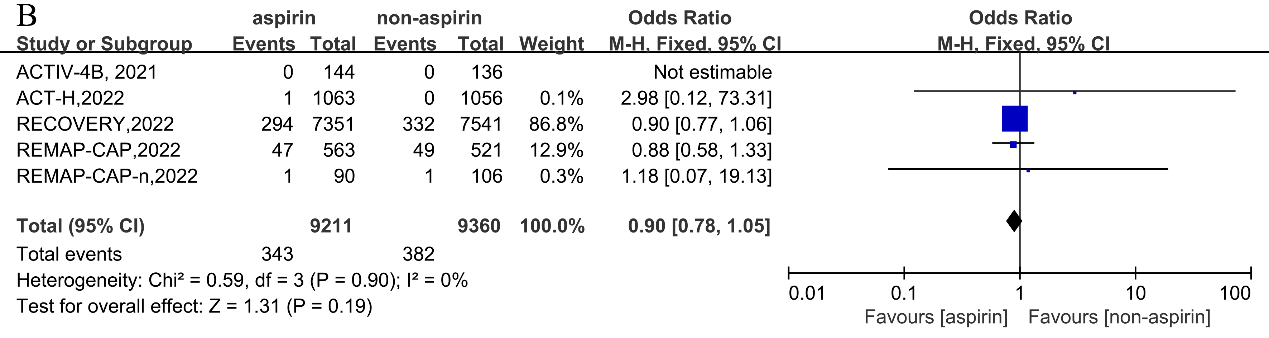


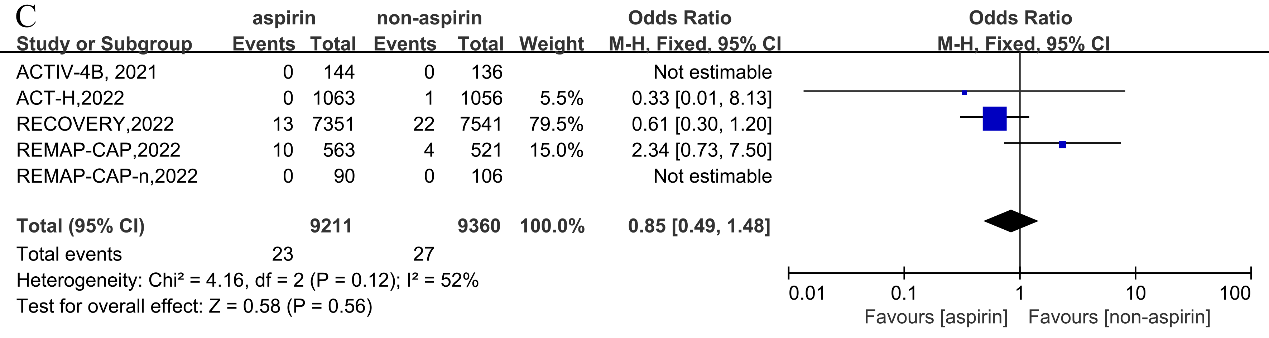


D


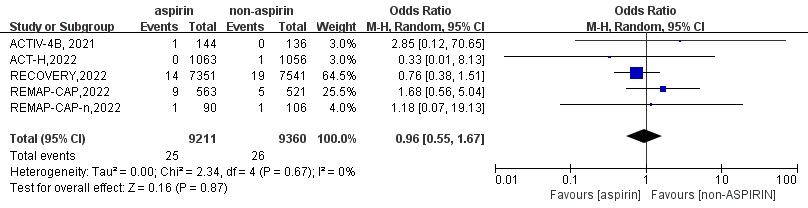


sFigure 4. Forest plots of asprin treatment vs. non-asprin treatment for being discharged alive. CI= confidence intervals; M-H= Mantel-Haenszel; IV= inverse variance


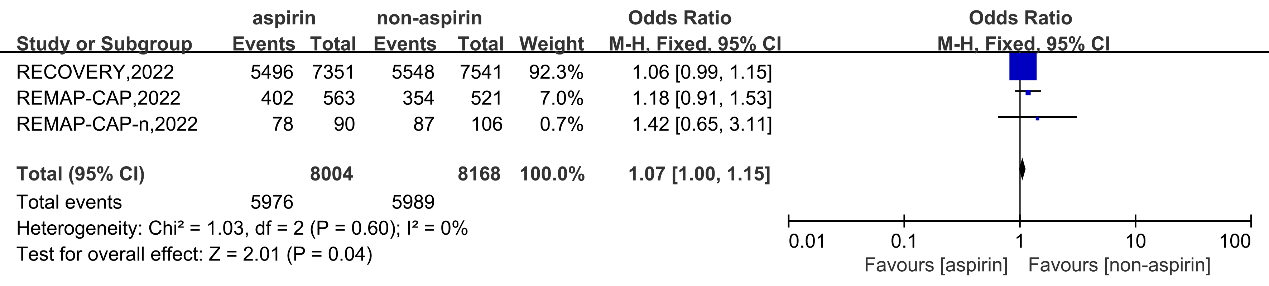


sFigure 5. Forest plots of asprin treatment vs. non-asprin treatment for acute kidney injury. CI= confidence intervals; M-H= Mantel-Haenszel; IV= inverse variance


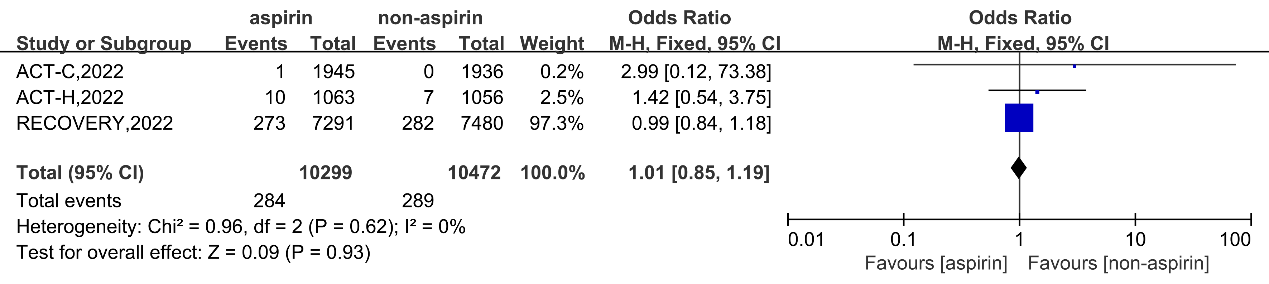

Supplement: Supplemental Information 6 [file peerj-13-19466-s006.doc]
